# Applying the Social Vulnerability Index as a Leading Indicator to Protect Fire-Based Emergency Medical Service Responders’ Health

**DOI:** 10.3390/ijerph18158049

**Published:** 2021-07-29

**Authors:** Emily J. Haas, Alexa Furek, Megan Casey, Katherine N. Yoon, Susan M. Moore

**Affiliations:** 1 National Institute for Occupational Safety and Health, National Personal Protective Technology Laboratory, Pittsburgh, PA 15236, USA; qet8@cdc.gov (A.F.); qek7@cdc.gov (K.N.Y.); sme6@cdc.gov (S.M.M.); 2 National Institute for Occupational Safety and Health, National Personal Protective Technology Laboratory, Morgantown, WV 26505, USA; ydg7@cdc.gov

**Keywords:** coronavirus, emergency management, risk management, social vulnerability index, firefighter, logit regression, occupational stress, relative weights, total worker health, emergency medical services

## Abstract

During emergencies, areas with higher social vulnerability experience an increased risk for negative health outcomes. However, research has not extrapolated this concept to understand how the workers who respond to these areas may be affected. Researchers from the National Institute for Occupational Safety and Health (NIOSH) merged approximately 160,000 emergency response calls received from three fire departments during the COVID-19 pandemic with the CDC’s publicly available Social Vulnerability Index (SVI) to examine the utility of SVI as a leading indicator of occupational health and safety risks. Multiple regressions, binomial logit models, and relative weights analyses were used to answer the research questions. Researchers found that higher social vulnerability on household composition, minority/language, and housing/transportation increase the risk of first responders’ exposure to SARS-CoV-2. Higher socioeconomic, household, and minority vulnerability were significantly associated with response calls that required emergency treatment and transport in comparison to fire-related or other calls that are also managed by fire departments. These results have implications for more strategic emergency response planning during the COVID-19 pandemic, as well as improving Total Worker Health^®^ and future of work initiatives at the worker and workplace levels within the fire service industry.

## 1. Introduction

Many fire departments provide not only firefighter but also emergency medical services (EMS). These fire-based EMS responders answer calls that deploy them to locations with limited knowledge and information, putting them at an increased risk for occupational exposure to infectious diseases and other injuries [1]. Consequently, these essential workers have been positioned at a critical intersection of public health, public safety, and healthcare systems [2,3,4]. To illustrate these intersecting roles, in 2018, just over 64% of fire department calls were for medical emergencies [5]. Another analysis determined that, when combining career and volunteer firefighters in the U.S., these workers respond to an average of one structure fire every other year, where most of their calls are emergency medical and incident responses [6].

Fire-based EMS responders’ occupational risks and barriers to proactive safety efforts have become even more apparent during the COVID-19 pandemic. First, these essential workers continued to face substantial occupational exposure while lifting, moving, and carrying patients [7,8]. Although less information is known about infection rates, a survey of a Fire Department in California identified that 48% of fire-based EMS responders had experienced likely or confirmed contact with patients with COVID-19, while another study concluded that New York City firefighters and EMS responders were 15 times more likely to be infected than the general public [9,10].

There are various reasons that fire-based EMS responders may be more susceptible to SARS-CoV-2 infections including long work shifts that may be 24 h or more, limitations in their ability to practice physical distancing both with their coworkers and during patient interactions, and possible shortages in personal protective equipment (PPE) [11]. Similarly, the CDC [12] indicates that fire-based EMS responders are at an increased risk of exposure based on unique factors, including constantly changing settings, being in an enclosed space during patient transport, needing to make quick decisions with limited information, and caring for a range of patients with different healthcare resources. Due to these varying elements that may contribute to exposure in any given scenario, it is imperative to identify any external factors or combination of factors that may help fire-based EMS responders and fire departments better predict and proactively mitigate occupational risks on the job.

Another barrier to addressing fire-based EMS responders’ unique risks both prior to and during the COVID-19 pandemic is the paucity of surveillance data at the national level [7], with systems capturing different data points. Studies have highlighted multiple surveillance systems that capture firefighter deaths, each with different exclusion/inclusion criteria [13,14]. Examples of national surveillance systems within firefighting include those overseen by the National Fire Protection Association (NFPA), the International Association of Fire Fighters, the Bureau of Labor Statistics’ Census, and case data from NIOSH’s Fire Fighter Fatality Investigation and Prevention Program. There are also individual computer-aided dispatch systems that are unique to a municipality and fire department. These varying systems make it difficult to collect accurate and timely information, much less be able to inform prevention by predicting potential incidents. Researchers concluded that, although fatality information may be consistent, data estimating risks were disparate [14]. Therefore, research examining the utility of existing datasets, including the ability of the data to predict scenarios in which fire-based EMS responders face increased risks of exposure to SARS-CoV-2, is important.

Poplin and colleagues [15] suggest that the diverse hazards experienced in the fire service should be accommodated through an adaptable approach to managing risks. However, the response to addressing fire-based EMS responders’ risks to improve worker safety, health, and well-being has been slow in coming for several reasons, even prior to the COVID-19 pandemic. As indicated earlier, fire-based EMS responders have a dynamic, mobile workplace which contributes to additional job stressors, including burnout, fatigue, stress, reduced performance, and negative health outcomes [16,17,18,19,20]. The same outcomes are true for other public safety occupations such as law enforcement [21,22]. This worker population routinely faces unknown risks; however, the COVID-19 pandemic allowed an opportunity to study the prevalence of this common risk factor to improve efforts around emergency planning and response. Research has advocated for the importance of understanding the locations of at-risk groups to help allocate resources for communities efficiently before, during, and after emergencies [23]. However, this information is less often used to support fire-based EMS responders’ own decision making during a response.

### 1.1. Total Worker Health^®^ Approach to Mitigating Fire-Based EMS Responders’ Risks

Due to these changing work environments that also come with surveillance challenges, traditional approaches to workplace safety may not be as effective within public safety occupations. Rather, a more holistic approach, such as the use of a Total Worker Health^®^ (TWH) framework, may be beneficial [24]. This paper discusses leading indicators within the realm of NIOSH’s TWH^®^ framework [25] to advance future of work initiatives [26] for the fire services. According to Smith and colleagues, fire-based EMS workers are the “embodiment of high-risk workers, facing extreme hazards that pose not only physical risk but psychological and psychosocial risk” [24] p. 194. Occupational connection to TWH^®^ principles were argued prior to the COVID-19 pandemic, to facilitate a focus on protecting workers facing extreme hazards [27,28]. Now, in response to the pandemic, a TWH^®^ framework and its corresponding elements may be even more applicable to guide workplace solutions to enhance worker health and well-being.

#### 1.1.1. Health Leading Indicators

During the COVID-19 pandemic, the basic principles of risk and emergency management have remained the same, with guidelines serving to help organizations adapt processes and practices to protect workers [29]. However, the pandemic has likely served as an impetus for organizations to integrate and promote additional practices to improve operational readiness and mitigate negative worker outcomes [29,30,31]. Although this represents a positive shift in priorities, organizations that experience frequent changes to the workplace should continually search for new leading indicators to help prevent and manage emergencies [32]. The CDC has discussed the importance of emergency responders knowing vulnerabilities in communities so they can anticipate needs and provide information to people at the right time [23]. Knowing this information can also help these responders with their own proactive planning to allocate resources and protect themselves.

Leading indicators are proactive, predictive, and preventative measures that provide insight into the future [33,34]. More specifically, health leading indicators refer to “the measures and actions that an organization can take to predict success in worker health outcomes and predict the operation of an organization’s health and wellbeing programming” [35], p. 3. Others discuss health leading indicators as insights into public health concerns that can promote behaviors and processes to improve worker health [36,37,38]. Such ongoing data points may help prevent illness and advance worker health and well-being. The development of CDC’s Social Vulnerability Index (SVI), for example, has helped local, state, and federal organizations identify and target vulnerable locations. However, the SVI has not been applied as a leading indicator to inform potential TWH^®^ and well-being approaches within the fire service industry.

#### 1.1.2. SVI as a TWH^®^ Leading Indicator

Research has recognized and documented the impact of social factors on public health. Results have shown that socially vulnerable populations, also referred to as disproportionately affected populations, are more likely to be adversely affected during emergencies [39,40,41,42,43,44,45,46]. To assist in emergency preparedness efforts, reliable surveillance is critical. The Agency for Toxic Substances and Disease Registry (ATSDR) developed the SVI to understand any potential correlations with community outcomes [47]. For example, limited research has used SVI to identify counties that were hot spots for COVID-19, e.g., [48]. The SVI uses 15 social factors from 4 main themes to create a vulnerability score between 0 (least vulnerable) and 1 (most vulnerable). These four themes include (1) socioeconomic status; (2) household composition/disability; (3) minority status/language; and (4) housing type/transportation.

SVI has been used at the state level to customize and localize response efforts. For example, Georgia and North Carolina have both developed state-specific adaptations of the SVI with their health departments [23]. Additionally, fire departments acknowledge the importance of understanding community profiles, including social vulnerability, to accurately assess risks prior to a response. For example, Ohio’s Columbus Fire Department, references SVI as an important tool to help officials and responders identify communities that need additional support [49].

Despite the use and connections made between SVI and negative health outcomes for communities and their residents, research has yet to directly focus on the occupational risks for the essential workers who serve these communities. To that end, this study applied SVI as a predictive health leading indicator to mitigate risks for fire-based EMS responders. Emergency preparedness and response has been recognized as an issue impacting all topics within NIOSH’s future of work priorities [50]. Understanding the direction of this relationship may have implications for preserving fire-based EMS responders’ safety and health during response activities not only during the COVID-19 pandemic but also for improving TWH^®^ initiatives within the fire services.

### 1.2. Research Questions

In this study, NIOSH researchers focus on how to effectively use existing data sources to identify hazardous patterns and mitigate worker risks. Specifically, using data received from three fire departments during the COVID-19 pandemic, NIOSH researchers merged and examined the utility of CDC’s SVI as a leading indicator to improve future of work initiatives in the fire service industry. Results elucidate ways that current surveillance data can be used and improved, and how the incorporation of a TWH^®^ approach can help identify and implement organizational-level interventions.

Research questions for this study are as follows:

RQ1: Do the four SVI themes predict fire-based EMS responders’ potential exposure to SARS-CoV-2 during response calls?
RQ2: Do the four SVI themes predict the types of calls to which fire-based EMS responders are deployed (i.e., EMS, fire, and other)?

Based on the results of these questions, this paper seeks to understand how fire departments and comparable organizations can use SVI to address future of work initiatives within a TWH^®^ framework.

## 2. Materials and Methods

In May 2019, NIOSH executed several collaborations to advance its public safety research portfolio. Through Research Collaboration Agreements (RCAs), NIOSH supported fire department subscriptions to the International Public Safety Data Institute’s (IPSDI) National Fire Operations Reporting System (NFORS). The NFORS application is comprised of two data modules that report details of emergency response calls:

(**1**) Computer-aided dispatch (CAD) module. The CAD extracts fire department operations information that is automated within municipality reporting systems. These data elements include the type of call, duration of call, exposures during the call, and specific resources deployed to the call (e.g., ladder truck, ambulance).
(**2**) Individual exposure module (i.e., career diary). A worker’s career diary is manually filled in by a worker after a completed response using either a smart phone or desktop. Within this application, workers input information about potential exposures (e.g., fire, hazmat, chemical, or biological) and personal protective equipment (PPE) worn during the incident (e.g., turnout gear, disposable face shield, or respirator).

### 2.1. NFORS Data Variables

When the COVID-19 pandemic began, NIOSH leveraged its existing relationship with IPSDI, the administrator of NFORS, to further understand aspects of incident reporting and response during COVID-19. Through the already-established RCAs with fire departments and data use agreements with IPSDI, NIOSH researchers continued to receive emergency call response data in monthly batches that contained call details from three fire departments in Ohio, Massachusetts, and New York. Hundreds of variables are included in these datasets, but the variables used in the current study are debriefed below.

#### 2.1.1. NFORS Self-Reported Potential Exposure to SARS-CoV-2

The Occupational Safety and Health Administration (OSHA) defines a potential occupational exposure as a “reasonably anticipated skin, eye, mucous membrane, or parenteral contact with blood or other potentially infectious materials that may result from the performance of an employee’s duties” [51]. Within the NFORS exposure module, such types of biological and chemical exposures are collected. Shortly after the COVID-19 pandemic started, IPSDI added a “Contagious Emergency/COVID-19” exposure response option [52]. The respective CAD systems were also modified to designate COVID-19 call types in CAD dispatch logs as “Contagious Emergency”. This addition to the incident reporting system ensured that departments could easily filter for and track COVID-19 incidents. This addition also allowed NIOSH researchers to appropriately filter and analyze data by exposure type.

#### 2.1.2. NFORS Call Type

The National Fire Incident Reporting System (NFIRS) provides a standardized system that fire departments can use to describe and ultimately code the details of each incident response [53]. Following suit with NFIRS, the IPSDI NFORS application groups these incidents into large categories of “EMS”, “Fire”, and “Other” based on the incident description and who responded to the call (i.e., fire unit only or fire-based EMS unit). Fire incidents are self-explanatory and can be any indoor or outdoor fire, including a fire alarm. EMS calls vary and can entail a motor vehicle incident, trauma, or health event such as a stroke or heart attack. Finally, service calls, good intent calls, and false alarms are often coded as “Other” call types [53] in the database. Call type was used as a dependent variable in an additional analysis to further understand the use of SVI as a valid leading indicator within the fire services.

### 2.2. Other Imported Data Variables

#### 2.2.1. Monthly Average of COVID-19 New Cases

Due to the self-reported cases of exposure to SARS-CoV-2, NIOSH researchers also added and controlled for the monthly average of COVID-19 new cases by county. The data were obtained from the New York Times (NYT) public data source hosted on GitHub [54]. Using the NYT data, researchers were able to match each county for which data were received with the averaged new daily cases added.

#### 2.2.2. Social Vulnerability Index

The SVI data are publicly available from the CDC’s website, which is not based on clinical or patient data. Data for all SVI themes and subtheme components were downloaded and used for analysis. CDC’s SVI values are at the census tract, not the county level. The 2016 data [55] were downloaded from three states based on the response area covered by the participating fire departments. Participating fire departments were from Ohio, Massachusetts, and New York. After downloading the data, researchers matched the NFORS call data by census tract to the CDC’s SVI data, which ranks census tracts from 0 to 1, where higher percentile rankings indicate greater social vulnerability. Call data were aggregated to the census tract level to establish the association of social vulnerability with SARS-CoV-2 exposure during emergency response calls. The SVI themes and factors are outlined in Table 1.

### 2.3. Sample

From March through September 2020, data from 162,766 incident responses were received from three fire departments. After excluding for missing data, there were 161,948 incident responses. Of the three departments included in this study, 26.7% of the incidents were from one department; 69.7% from the second; and 3.6% from the third. Of these incidents, 99.4%, were considered “urban” in terms of population density and the remaining 0.6% were considered “rural.” The lowest number of incidents was reported in April (11.9% of the sample) and the highest number in July (16.0%). Regarding days of the week on which incidents were responded to, calls were evenly dispersed, with the lowest number of incidents reported on Saturday (13.7% of the sample) and the highest number of incidents reported on Monday (14.8%). Within the sample, 63.3% of the calls were coded as EMS; 30.4% as Fire; and 6.3% as Other.

### 2.4. Analysis

As stated in the research questions, the main interest was determining the association between SVI and potential exposure to SARS-CoV-2 and call type. Consequently, it was important to control for confounding variables to reduce omitted variable bias. To help account for variable selection, researchers controlled for several variables, some of which were discussed in the above section and are highlighted again below:

- COVID-19 new cases: New cases of COVID-19 were controlled for in the analysis because the prevalence of COVID-19 infections in an area might affect the call type (i.e., higher rates of EMS calls) and subsequent potential exposure to SARS-CoV-2.
- Fire department/state: Knowing infection rates could be different by state, based on the time of the study, researchers aimed to capture regional effects (by state/department) by creating fire department dummies for use in the analyses.
- Month: Knowing infection rates could vary over time, researchers aimed to capture seasonal effects (by month) by creating time dummy variables.
- Population: If a fire department covers an area with a larger population, fire-based EMS responders may be more likely to be exposed to SARS-CoV-2, as well as receive certain types of calls, in comparison to departments who serve smaller populations, making population an important control variable.

Prior to performing the analyses, researchers examined the data. A root-mean square error (RMSE) and R2 were compared using a training sample of 80% and the remaining 20% as the designated test sample. Results were within the prescribed guidelines. Tolerance was also measured for each theme, and all were within an acceptable range between 0.32 to 0.69. Researchers concluded that multicollinearity was not a significant problem within the dataset and that the overall fit of the model was satisfactory.

#### 2.4.1. SVI and Exposure to SARS-CoV-2

A binomial logit model was used to explain the effect of the four SVI themes on the probability of SARS-CoV-2 exposure during response calls controlling for total population, COVID-19 new cases, and department, as follows:

(1) $$\begin{array}{c} Logit\left(Y\right)=Ln\left(\frac{p}{1-p}\right)=\beta_{0}+\beta_{1}THEME1+\beta_{2}THEME2+\beta_{3}THEME3+\\ \beta_{4}THEME4+\beta_{5}TOTPOP+\beta_{6}COVIDnewcases+\beta_{7}Department \end{array}$$

After the logistic regression, a pair-wise comparison was conducted to assess the difference between New York and Ohio (Massachusetts was set as the baseline in the regression model). Additionally, due to the sample size, researchers performed a relative weights analysis [56,57] to determine the relative contribution of each predictor towards explaining variance in the criterion variable. Researchers partitioned R-squared into pseudo-orthogonal portions, with each portion representing the relative contribution of one predictor variable. The rescaled relative weights sum to 100% and the raw relative weights sum to the observed value of R-squared.

#### 2.4.2. SVI and Types of Calls

To further understand the predictive utility of SVI, a multiple logistic regression was used, controlling for total population, COVID-19 new cases, and department. Further, relative risk ratios were determined through these analyses. Due to the sample size, researchers performed a relative weight analysis, as follows:

(2) $$\begin{array}{c} \mathrm{Ln}\left(\frac{P\left(\mathrm{calltype}=\mathrm{FIRE}\right)}{P\left(\mathrm{calltype}=\mathrm{EMS}\right)}\right)=\beta_{10}+\beta_{11}\mathrm{THEME}1+{\;\beta }_{12}\mathrm{THEME}2+{\;\beta }_{13}\mathrm{THEME}3+\\ {\;\beta }_{14}\mathrm{THEME}4+{\;\beta }_{15}\mathrm{TOTPOP}+{\;\beta }_{16}\mathrm{COVIDnewcases}+\beta_{17}\left(\mathrm{department}=\mathrm{NY}\right)+\\ \beta_{18}\left(\mathrm{department}=\mathrm{OH}\right) \end{array}$$

$$\begin{array}{l} \mathrm{Ln}\left(\frac{P\left(\mathrm{calltype}=\mathrm{OTHER}\right)}{P\left(\mathrm{calltype}=\mathrm{EMS}\right)}\right)\\ \;=\beta_{20}+\beta_{21}\mathrm{THEME}1+{\;\beta }_{22}\mathrm{THEME}2+{\;\beta }_{23}\mathrm{THEME}3+{\;\beta }_{24}\mathrm{THEME}4\\ \;+{\;\beta }_{25}\mathrm{TOTPOP}+{\;\beta }_{26}\mathrm{COVIDnewcases}+\beta_{27}\left(\mathrm{department}=\mathrm{NY}\right)\\ \;+\beta_{28}\left(\mathrm{department}=\mathrm{OH}\right) \end{array}$$

where

β_10_: intercept;
β_11_: A one-unit increase in the variable Theme1 is associated with the change in the log odds of having FIRE call vs. EMS call in the amount of β_11_;
β_21_: A one-unit increase in the variable Theme1 is associated with the change in the log odds of having OTHER call vs. EMS call in the amount of β_21_;
β_17_: The log odds of having FIRE call vs. EMS call is associated with a change by β_17_ if moving from department MA to NY;
β_28_: The log odds of having OTHER call vs. EMS call is associated with a change by β_28_ if moving from department MA to OH.

## 3. Results

### 3.1. Descriptive Statistics

Table 2 shows the descriptive statistics for the variables within the current study. Table 3 shows thematic vulnerability by SARS-CoV-2 exposure and emergency response call type, and Table 4 shows thematic vulnerability by exposure to SARS-CoV-2; department SVI mean plots for these variables are in Appendix A.

### 3.2. RQ1 Logit Regression for SVI and SARS-CoV-2 Exposure

In this analysis, logistic regression was performed on the potential exposure to SARS-CoV-2. All four SVI themes significantly predicted the probability of fire-based EMS responders’ exposure to SARS-CoV-2 when responding to a call. Holding other variables constant, an increase in Themes 2, 3, and 4 was positively associated with exposure probability to SARS-CoV-2. Specifically, a one-unit increase (0.01 in RPL ranking percentile) in social vulnerability had the following impact (controlling for other variables):

- Theme 2—household composition increased the odds of exposure to SARS-CoV-2 by 49.3%;
- Theme 3—racial/ethnic minority/language increased the odds of exposure to SARS-CoV-2 by 94.4%;
- Theme 4—housing/transportation increased the odds of exposure to SARS-CoV-2 by 33.6%.

Regarding Theme 1, socioeconomic status was negatively associated with exposure to SARS-CoV-2. A one-unit increase in Theme 1 decreased the odds of having an exposure to SARS-CoV-2 by a factor of 0.210 (odds ratio = 0.810, *p* = 0.004), or 19%. Table 5 shows a full summary of results. Predicted probability plots are in Appendix B.

As indicated, due to the large sample size, a relative weights analysis was completed to ensure the integrity of the data in explaining potential exposure to SARS-CoV-2 during responses. The results demonstrated that the number of COVID-19 new cases had the heaviest weight of importance, meaning the number of new cases explained potential SARS-CoV-2 exposure the most (68.23%), followed by department (17.12%). The remaining 14.7% of the variance was explained by the SVI themes. Minority index (Theme 3) had the heaviest weight, followed by housing/transportation vulnerability (Theme 4).

### 3.3. RQ2: Multiple Logistic Regression for SVI and Call Type

Researchers further examined the utility of SVI as a leading indicator within the fire services. In this analysis, multinomial regression was performed on different fire-based EMS call types (EMS, Fire, and Other). Controlling for total population, COVID-19 new cases, and department, a higher SVI for Theme 1 was significantly associated with more EMS calls than non-EMS calls (i.e., Fire and Other). This was the same for Themes 2 and 3. For Theme 4, however, a higher SVI was associated with more EMS calls compared to Fire but was the opposite for other calls. Table 6 shows the multiple logistic regression results.

A relative weights analysis on the call type showed that department was the strongest predictor. After department, the four SVI themes had significantly higher relative weights than the other control variables (total population and COVID-19 new cases) at 22.8% of the variance in explaining the call type. Theme 2 had the heaviest weight, followed by Theme 3, Theme 4, and Theme 1.

## 4. Discussion

Using potential exposure to SARS-CoV-2, this study applied CDC’s SVI as a leading indicator of fire-based EMS responders’ potential health outcomes during response calls, rather than as a measure of emergency and disaster preparedness. Previous findings [47,48,58,59,60] have shown that COVID-19 disproportionately affects certain areas more than others. The current results show that fire-based EMS responders who answer calls to these areas with a higher SVI can experience higher incidents of exposure to SARS-CoV-2. These risks are likely not only specific to occupational exposure to SARS-CoV-2 but also to other hazardous conditions faced during unknown emergency calls, requiring more patient interaction, which was also predicted by the SVI themes. Therefore, these results may have implications for emergency response planning, including resource allocation and supply distribution.

Specifically, this analysis provides evidence that adequate supplies and training are critical to fire-based EMS responders who serve populations with higher vulnerability. Consequently, results can be incorporated into fire-based EMS responders’ approaches to prepare for, respond to, and communicate with patients. This information also provides a new level of situational awareness for advancing NIOSH’s future of work initiative, including ways to control risks, monitor changes, and connect trends in the workplace, work, and workforce. NIOSH’s future of work initiative encourages the use of a TWH^®^ framework to collaborate on improving policies, programs, and practices [26]. To that end, this discussion focuses on several TWH^®^ elements, as emphasized within NIOSH’s future of work initiative, in which the identification of SVI as a leading health indicator can be used to improve fire-based EMS responder health outcomes and well-being.

### 4.1. Health Promotion Interventions That Engage Workers on and off the Job

Both research questions showed that areas with higher social vulnerability were predictive of fire-based EMS responders’ direct interaction with patients through EMS calls and, not surprisingly, potential exposure to SARS-CoV-2. Consequently, it is important to emphasize research related to social determinants of health (SDOH) [61]. Addressing SDOH through the development of health promotion resources and interventions can indirectly benefit fire-based EMS responders who serve these communities, in addition to the residents. NIOSH has proposed such integrations of occupational safety and health protections in communities and workplace health promotion activities [25] to improve efficiency, resource management, and worker health [62]. These health promotion efforts such as safe work locations, worker housing, and community safety recognize the blurred boundaries between work, home, and community life [63]. However, there is a gap in empirical interventions within the fire service [64], making future research-to-practice in this area important.

Also, engaging workers in the development of health promotion resources and interventions is a key element of TWH^®^ [25]. If fire-based EMS responders are involved in community–workplace intervention design, it is more likely that proposed initiatives will fit their workplace context, improve organizational learning, and improve sense of ownership and subsequent participation [65,66,67,68]. The current SVI results provide space for a variety of targeted interventions that can involve fire-based EMS responders, including the following:

- Incorporate and update SVI as a health leading indicator into safety and health programs around hazard recognition and risk assessment (for examples of such indicators, see [33,34,38]). As previously mentioned, some fire departments have made this addition to their risk assessment programs already e.g., [49]. This mindful addition will allow SVI to be included and acknowledged in organizational audits of fire departments’ safety and health management systems.
- Incorporate SVI information into health promotion/wellness programs within the fire services, including easy-to-read materials on how psychosocial factors influence workers’ health outcomes, for examples, see [69,70].
- Develop and include educational training materials to maintain safety and health when responding to areas with higher social vulnerability. For example, fire departments can work with their public health departments to develop and provide plain-language, culturally sensitive, and relevant public health messaging tailored to community needs. Regarding COVID-19 specifically, educational materials could be multi-lingual and contain known risk factors for adverse outcomes [71]. Fire-based EMS responders can have these resources with them during emergency response calls to help educate the community and minimize communication gaps with community residents during good intent or other nonlife-threatening calls.

These efforts could be particularly helpful, as one study determined that inconsistent communication is associated with higher SVI [72]. Therefore, an advantage to working with public health departments during health promotion efforts is that all local public safety and health leaders can communicate the same message in the same way within their communities.

Taking NIOSH’s future of work perspective into account, there are additional aspects to consider. Specifically, worker groups within the fire services that are disproportionately vulnerable to certain health risks should be involved in any type of program design and implementation [27,73]. For example, fire-based EMS responders who work rotating or night shifts and are more susceptible to fatigue, as well as older workers who are more susceptible to cardiovascular health issues, could be asked to give input regularly. Their involvement is necessary to identify job characteristics and work processes that impede participation in health promotion activities during emergency response calls [74]. Additionally, their feedback can help identify resource allocation issues before making larger changes to the workplace [24].

### 4.2. Improvement of Data Surveillance at the Organizational and Policy Levels

Another key element of TWH^®^ that is also discussed in NIOSH’s future of work initiative is the use of data-driven results to improve outcomes. The CDC has discussed the availability of timely surveillance data as a primary goal in its future of work data modernization initiatives [75]. Data modernization also includes the ability to use artificial intelligence (AI) and machine learning techniques on surveillance data. Regarding firefighter surveillance data, researchers have argued for the necessity of using AI to account for worker emotions during emergency responses, which can help with data objectivity and accurate risk identification [76]. These results demonstrate that not only improvements in data surveillance but also the application of those data by the fire service can be improved to identify latent, health leading indicators. The value of better surveillance from two perspectives is discussed: increased AI capabilities to predict future incidents and increased risk assessment capabilities on the ground to prepare workers.

#### 4.2.1. Emergency Call Surveillance Data for Machine Learning

There is a critical need to advance occupational safety and health surveillance [50]. Improved data can allow researchers, practitioners, and organizations to identify groups at higher occupational risk and subsequently tailor interventions [73,74]. Specific to emergency response data, previous research has argued that a strained 911 system is responsible for inaccurately understanding resources needed during response calls [77], including the need for what PPE to bring and use during a response call [78]. In these referenced studies, participating EMS workers and firefighters discussed the need for improvements to the 911 data catalogue system to assist with accurate triage efforts, targeting the use of EMS resources, updating continuing education based on job demands, and improving a social safety net to address the persistent needs of populations of older adults (aged ≥65 years) and people with lower incomes [77].

The current results provide two recommended action items to improve surveillance efforts. First, SVI, as a publicly available data point, can be integrated and regularly updated in CAD systems and used as more than a measure for emergency response. Second, SVI can also be used as an objective data point in emergency call logs to provide advanced surveillance opportunities. Research has identified the value in updating computerized work systems for improving policy and practice standards [79], and this addition may inform such standards. Additionally, although machine learning has greatly advanced, there are still issues related to algorithm bias and trust in the results [80]. It is possible that, over time, using SVI as a leading indicator with other details of emergency response call logs can help ensure that responders have the training and resources needed to protect themselves from potential exposures, including SARS-CoV-2, during every response call.

To illustrate, using the original data codes provided to NIOSH, regression models showed that higher SVI was associated with EMS calls over Fire and Other calls (except for one theme). To examine whether this trend remained, researchers further coded and created two new variables for response calls that were received in large quantities (i.e., automobile calls and hazmat calls). Using the same analyses as in RQ2, emergency calls involving automobile incidents were predicted by a higher SVI in Theme 1 and 2 in comparison to the remaining EMS calls. This small case example shows the value of improved surveillance data in the fire services and that objective data points are needed to inform higher-level predictive analytics. This barrier has also been referenced and discussed by others, e.g., [53,81], but it is necessary to improve it as the future of work changes.

#### 4.2.2. Response Planning and Resource Allocation

It is possible that integrating SVI into more systematic surveillance efforts can elucidate some of the inconsistencies among available tools that are used to identify, plan for, and respond to areas of higher risk for workers [82]. All employees having access to available and useful resources is important, as a challenge regarding the future of work is ensuring the equitable distribution of benefits and risks that accompany phenomena such as nonstandard work arrangements [50]. It may be beneficial for fire departments to incorporate social vulnerability assessments into emergency management practices, including needs assessment tools and preparedness checklists. For example, research has indicated that SVI can be used to decide how many emergency personnel should be sent to assist during a response [49]. Using checklists has been deemed an appropriate method for involving fire-based EMS workers in needs assessment efforts [83,84]. They can also help address the needs of vulnerable workers through increased transparency [85]. Therefore, using SVI data to update needs assessment tools regularly may enhance public health planning and response efforts for these workers, allowing fire-based EMS responders to identify communities that may require more resources during a call (e.g., additional PPE, apparatus, or workers), or to have a more accurate understanding of the type of call to which they may be responding.

In the current results, a higher SVI in Themes 2, 3 and 4 were predictive of exposure to SARS-CoV-2. Fire departments can use these results to update aspects of not only COVID-19 health and safety plans to protect employees but also emergency preparedness plans for future occupational exposure risks. When deployed to areas with a higher SVI in these themes, EMS strategies can include maintaining familiarity with the CDC’s COVID-19 resources for firefighters and EMS providers and employing recommended strategies such as wearing the recommended PPE or providing facemasks or cloth coverings to patients for source control if they do not have any [12]. A similar strategy has worked well in a recent field-based study, where an EMS location updated its dispatch systems to gather high- and low-risk criteria to determine whether an alert “PPE advised” should be provided to the EMS responders for a particular call [8]. SVI data can help inform part of this decision making in the future.

### 4.3. Limitations

This study demonstrated the utility of SVI as a health leading indicator in the fire services, identifying opportunities in future surveillance efforts, potential resources for adequate emergency response planning and supply management, and possible updates to community-organizational health promotion efforts in the areas to which fire-based EMS responders are deployed. These results suggest opportunities to make immediate changes to help protect fire-based EMS responders and provide implications for improving future of work initiatives. Although the suggested initiatives are promising, there are limitations to the data. First, researchers pointed to gaps in the current dataset around emergency system call coding, which must be considered. Additionally, potential SARS-CoV-2 exposure was self-reported, and it is unclear whether these potential exposures resulted in COVID-19 diagnoses amongst exposed responders. Researchers controlled for these data limitations by further exploring and recoding the call type variable and controlling for actual COVID-19 cases in the three departments included in the sample. However, even the controlled COVID-19 cases are limited due to issues around testing availability, so the true prevalence is still unknown. Therefore, future surveillance research is necessary to best understand these results.

Second, there are many external factors that make implementing some of these suggestions difficult and thus illustrate limitations in the feasibility of the results. For example, although prioritizing resources during higher-risk calls such as PPE use may be desired, the availability of such resources may make adherence to suggestions difficult [86]. Additionally, this study did not take the size of these departments into consideration when providing potential future of work initiatives using SVI. Previous research has discussed the impact that organization size can have not only on the resources available to develop safety and health programs but also the efficacy in being able to effectively implement them to protect worker safety, health, and well-being, e.g., [87,88]. Therefore, future research should address the feasibility of using SVI to inform safety and health decision making at different-sized fire departments.

## 5. Conclusions

Despite the limitations of the current study, the results show promise in the value of health leading indicators in the fire services, as well as how these indicators can be used to address challenges associated with the future of work. A result of improved leading indicator identification, particularly within emergency management, is that organizations and their employees are aware of and can adjust to hazards to prevent negative outcomes. This study was unique in that most occupational safety and health research focuses on safety leading indicators [38], whereas this analysis focused on health leading indicators to prevent occupational exposure and improve worker preparedness and well-being.

Research has argued that emergency management must use the right technology and tools to support work processes needed during dynamic response scenarios [32]. Opportunities for improved work processes were revealed in this study, including (1) health promotion activities and interventions that engage workers, (2) improved data surveillance methods, and (3) the development of accurate, regularly updated needs assessments. The CDC has discussed the importance of using and integrating data to guide improvements in communication plans, emergency preparedness, and to develop comprehensive pictures of groups with higher occupational risk [73,89]. The results of this study illustrate the value of using data to not only compile an accurate understanding of groups with higher occupational risk, but also ways to positively impact fire-based EMS responders’ health and well-being over time.

## Figures and Tables

**Table 1:** CDC SVI 2016 themes and subthemes [55].

SVI Theme	SVI Subthemes	Negative Impact
**Theme 1:** Socioeconomic status (SES)	Percentage of persons below poverty estimate, percentage unemployed estimate, per capita income estimate, and percentage with no high school diploma estimate	Often lack resources needed to comply with emergency preparedness instructions
**Theme 2:** Household composition/disability	Percentage of people ≥65 and ≤17 years old, percentage with a disability, and percentage of single-parent households	Issues with communication and comprehension of an emergency, subsequent barriers to mobility, and more likely to need financial support
**Theme 3:** Minority status/language	Percentage in racial/ethnic minority groups and the percentage who speak English less than well	Trouble understanding public health directives, culturally insensitive messaging
**Theme 4**: Housing/transportation	Percentage multiunit structures, percentage mobile homes, percentage crowding, percentage no vehicle, and percentage group quarters	Live in multi-unit structures or mobile homes in group quarters, lack vehicle

**Table 2:** Descriptive statistics.

Variables				
Continuous variables	*N*	Mean	SD	Description
Theme 1 (SES)	156,983	0.6086	0.3016	Percentile ranking of SES
Theme 2 (household/disability)	156,983	0.5220	0.3474	Percentile ranking of household composition/disability
Theme 3 (minority status/language)	156,983	0.6201	0.2287	Percentile ranking of minority status/language
Theme 4 (housing/transportation)	156,983	0.5843	0.2717	Percentile ranking of housing/transportation
Population	156,983	4504	2416	Total population of the geographic area
COVID-19 new cases	156,983	131	91.52	Monthly averaged COVID new cases of the specified geographic area (i.e., FIPS)
Categorical variables	*N*	percentage		
Potential SARS-CoV-2 Exposure				
	156,983			
Yes	8143	5.2%		
No	148,840	94.8%		
Department	156,983			
Massachusetts	41,917	26.7%		
New York	5692	3.6%		
Ohio	109,374	69.7%		
Call Type	156,983			
EMS	99,297	63.3%		
Fire	47,729	30.4%		
Other	9957	6.3%		

**Table 3:** Thematic vulnerability by SARS-CoV-2 exposure and emergency response call type.

	Theme 1 SES		Theme 2 Household/ Disability		Theme 3 Minority Status/Language		Theme 4 Housing/ Transportation	
Potential SARS-CoV-2 exposure	Mean	SD	Mean	SD	Mean	SD	Mean	SD
Yes	0.6122	0.2793	0.5195	0.3329	0.7032	0.2191	0.6605	0.2557
No	0.6084	0.3028	0.5221	0.3481	0.6156	0.2283	0.5802	0.2719
Call Type	Mean	SD	Mean	SD	Mean	SD	Mean	SD
EMS	0.6382	0.2933	0.5732	0.3326	0.6015	0.2223	0.5581	0.2724
Fire	0.5566	0.3103	0.4401	0.3554	0.6439	0.2362	0.6185	0.2644
Other	0.5631	0.3019	0.4038	0.3489	0.6917	0.2298	0.6825	0.2570

**Table 4:** Thematic vulnerability by exposure to SARS-CoV-2 and department.

	Massachusetts		New York		Ohio	
Yes potential exposure	3212	7.7%	1338	23.5%	3593	3.3%
No potential exposure	38,705	92.3%	4354	76.5%	105,781	96.7%
Total	41,917		5692		109,374	
Vulnerability	Mean	SD	Mean	SD	Mean	SD
Theme 1	0.5524	0.2978	0.4650	0.2687	0.6377	0.2995
Theme 2	0.3778	0.3579	0.3662	0.2344	0.5854	0.3286
Theme 3	0.7555	0.2070	0.7434	0.2027	0.5618	0.2128
Theme 4	0.7370	0.1967	0.7074	0.2855	0.5194	0.2700

**Table 5:** Summary of logit regression results.

	Est.	Std.Err	Pr (>|z|)	Odds Ratio	95% Confidence Interval	
					(2.5%)	(97.5%)
(intercept)	−4.020 *	0.063	0.000	0.018	0.016	0.0203
Theme 1 (SES)	−0.210 *	0.072	0.004	0.810	0.703	0.934
Theme 2 (household/disability)	0.401 *	0.052	0.000	1.493	1.349	1.653
Theme 3 (minority status/language)	0.665 *	0.080	0.000	1.944	1.663	2.274
Theme 4 (housing/transportation)	0.289 *	0.056	0.000	1.336	1.197	1.491
Department						
NY	0.869 *	0.042	0.000	2.383	2.196	2.585
OH	−0.807 *	0.033	0.000	0.446	0.418	0.476
Population	0.000 *	0.000	0.000	1.000	1.000	1.000
COVID-19 Cases	0.005 *	0.000	0.000	1.005	1.005	1.005

Pearson’s chi-sq test (predicted vs. observed): X^2^ = 25,326; df = 3511; *p* < 0.001; Pseudo R-squared: CoxSnell = 0.0407; Nagelkerke = 0.1215; McFadden = 0.1019; Log likelihood of each variable: *p* < 0.001; * *p* < 0.01.

**Table 6:** Multinomial logistic regression results.

		FIRE			OTHER	
	Coefficient	RRR	95% CI	Coefficient	RRR	95% CI
(Intercept)	1.222 *	3.3931	3.3706–3.4157	−0.385 *	0.6803	0.6797–0.6809
SVI themes						
Theme 1	−0.254 *	0.7754	0.7697–0.7811	−0.062 *	0.9403	0.9395–0.9412
Theme 2	−0.368 *	0.6919	0.6873–0.6964	−0.637 *	0.5287	0.5283–0.5291
Theme 3	−0.281 *	0.7550	0.7511–0.7589	−0.048 *	0.9529	0.9522–0.9537
Theme 4	−0.036 *	0.9644	0.9593–0.9694	0.312 *	1.3668	1.3658–1.3678
Department						
NY	−1.656 *	0.1909	0.1908–0.1910	−1.362 *	0.2561	0.2561–0.2562
OH	−1.959 *	0.14106	0.1403–0.1418	−2.520 *	0.08043	0.0804–0.0805
Total population	−0.00000	1	1.0000–1.0000	−0.00002 *	1	1.0000–1.0000
COVID-19 cases	−0.0002 **	0.9998	0.9997–1.0000	−0.0003 *	0.9997	0.9995–0.9998
Residual deviance	227314					
AIC	227350					

Notes: * *p* < 0.01; ** *p* < 0.05; RRR = relative risk ratios; AIC = Akaika information criterion.

**Figure A1:**
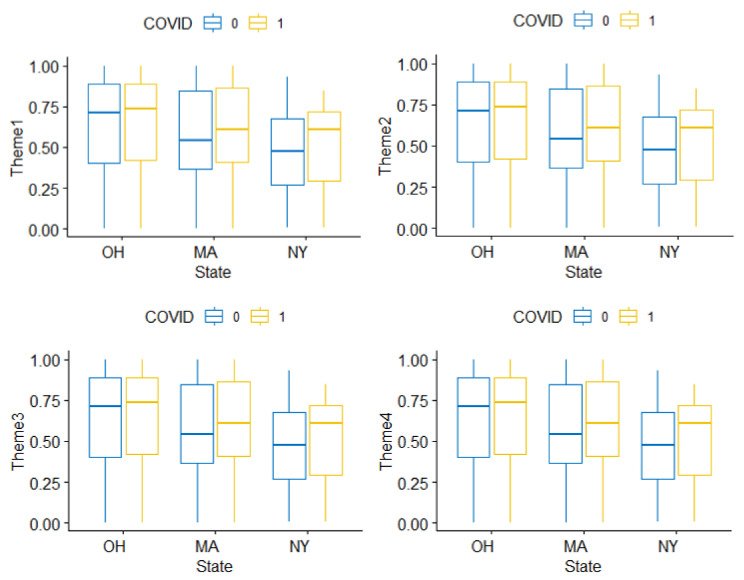
SVI mean plot with SE by exposure to COVID-19 and department.

**Figure A2:**
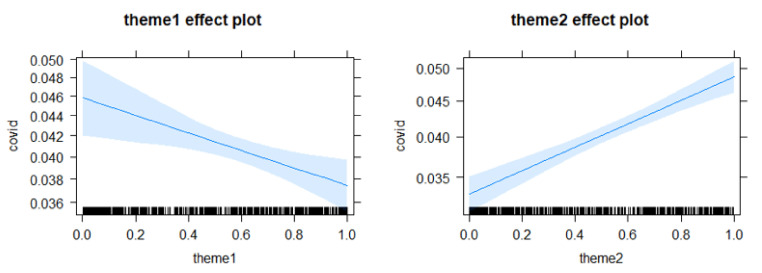

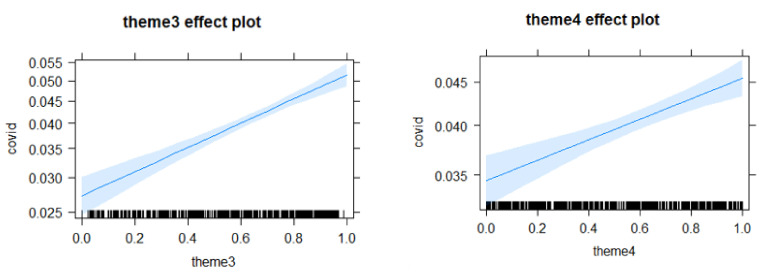
Predicted probability plots with 95% confidence interval by each predictor.

## Data Availability

Restrictions apply to the availability of these data. Data were obtained by a Research Collaboration Agreement with IPSDI and participating fire departments.
